# Lifestyle referral assessment in an acute cardiology setting: study protocol for a randomized controlled feasibility trial

**DOI:** 10.1186/1745-6215-14-212

**Published:** 2013-07-11

**Authors:** Kate M Hill, Rebecca EA Walwyn, Diana C Camidge, David M Meads, Jenni Y Murray, Greg Reynolds, Amanda J Farrin, Allan O House

**Affiliations:** 1 Leeds Institute of Health Sciences, University of Leeds, Leeds, UK; 2 Clinical Trials Research Unit, University of Leeds, Leeds, UK; 3 Leeds Teaching Hospitals Trust, Leeds, UK

**Keywords:** Cardiovascular risk factors, Lifestyle change, Randomized controlled trial

## Abstract

**Background:**

Lifestyle and behaviour change are important factors in the prevention of cardiovascular disease and reduction of premature mortality. Public health initiatives have focused on opportunities for healthcare staff to deliver lifestyle advice routinely in primary and secondary care but there is no consistent approach to onward referrals and the rate of uptake of advice remains low. We do not know if advice is more effective in supporting behaviour change when a systematic approach is taken that includes identification of barriers to change, directing patients toward services, referral to services, and feedback on outcome.

**Methods and design:**

This is a single-centre, randomized, unblinded feasibility trial in an acute hospital setting which aims to assess the feasibility of a definitive trial and provide proof of concept for the systematic delivery of individualized lifestyle advice in patients managed through an acute cardiology in-patient service.

Patients will be recruited before discharge and randomized to two groups. A control group will receive the usual lifestyle assessment and referral, while an intervention group will receive the usual assessment plus the new individualized lifestyle assessment and referral. The new assessment will inform assignment of each patient to one of three categories based on personal barriers to change. Patients may be referred to a formal lifestyle-change programme, through the ‘Leeds Let’s Change’ website, or they may be guided in self-management, using goal setting, or they may be assigned to a ‘deferment’ category, for reassessment at follow-up. These latter patients will be given a contact card for the ‘Leeds Let’s Change’ service.

**Discussion:**

Lifestyle change is an important mechanism for improving health and wellbeing across the population but there are widely acknowledged difficulties in addressing lifestyle factors with patients and supporting behaviour change. A systematic approach to assessment would facilitate audit and provide an indicator of the quality of care. The new assessment template has been designed to be quick and easy to use in practice and could, for example, be added to a primary care consultation or form part of a nursing discharge assessment in an acute setting.

**Trial registration:**

Current Controlled Trials ISRCTN41781196.

## Background

Cardiovascular disease is a global killer; it is a major cause of premature death and morbidity, and is responsible for over 4 million deaths in Europe annually [1]. It is clear that promoting good cardiovascular health has numerous benefits for society, including lower levels of morbidity, better wellbeing (or quality of life) and lower healthcare costs. Nevertheless, tackling the lifestyle behaviours associated with cardiovascular risk with interventions like smoking cessation, weight management, alcohol awareness and exercise is complex, as many factors, be they social and psychological, cultural or economic, may affect the individual’s willingness and ability to change [2-4].

Recent guidance from the UK’s National Institute for Health and Clinical Excellence (NICE) [5] states that attempts to change behaviour have been largely unsuccessful and that there is ‘…no strategic approach to behaviour change across government, the NHS or other sectors, and many different models, methods and theories are being used in an uncoordinated way.’ This, NICE concludes, is despite a mass of evidence from different disciplines on the theoretical basis for individual and societal-level behaviours and models of change. Divergent conceptual approaches and heterogeneous research methods make it difficult to merge evidence to inform the planning and design of services and their delivery.

Public health initiatives have focused on the opportunities to deliver lifestyle advice [3]. Healthcare staff in primary and secondary care routinely discuss lifestyle with patients but there is no consistent approach to the referral of patients to receive the support or services required for change. Moreover, the rate of uptake of lifestyle support services remains low [6]. In our previous work [4,7], we identified five individual barriers to behaviour change:

• Low mood, especially anxiety and depression.

• Poor knowledge about healthy lifestyles and poor education.

• Little support from family and friends.

• Financial and transport difficulties (where cost and travel is required for attendance at a lifestyle programme).

• A belief that changing lifestyle will have little impact on health.

The intervention to be tested will therefore consist of a new referral assessment for patients. The assessment will address these barriers to change and will be used to identify suitable services for patients.

### Financial incentives

Incentivizing individuals to encourage healthy living was recently discussed by the National Institute for Health and Clinical Excellence (NICE) Citizen’s Council [8]. This approach is already being implemented, for example, the ‘Help 2 Quit’ programme run by Shropshire NHS [9]; the ‘Give It Up For Baby’ run by NHS Tayside [10] offering incentives to pregnant women who quit smoking, and an NHS PCT in the south of England [11] offering financial incentives for weight loss.

Incentives are becoming more common in public health interventions for encouraging healthy behaviour, and some positive results have been reported for increasing weight loss [12], and levels of physical activity [13]. Nevertheless, while the use of incentives in this way may be a useful tool in encouraging behaviour change, recent reviews suggest that further research is needed to justify their widespread use [14,15]. This study will add to knowledge about the nature and scale of incentives that people deem acceptable.

This randomized feasibility trial is being conducted as part of the vascular research theme: Improving Prevention of Vascular Events (IMPROVE) in the National Institute for Health Research Collaboration for Leadership in Applied Health Research and Care (CLAHRC) for Leeds, York and Bradford. It will examine feasibility and provide proof-of-concept evidence for a larger-scale trial to test the hypothesis that the rate of uptake of lifestyle behaviour change can be increased by use of a systematic assessment that tailors the level of lifestyle support offered to individuals according to personal barriers and facilitators to lifestyle behaviour change. It is exploratory in nature rather than a scaled-down, pilot version of the main trial. The design of the main trial may be modified based on the findings of the feasibility trial.

The intervention (a lifestyle referral assessment) is based on evidence about key factors that are known to predict uptake of lifestyle behaviour change [2,4], and is specifically designed for use by care providers at the point where they might advise patients of lifestyle support services or refer patients to these support services. It incorporates a discussion of barriers and facilitators to changing lifestyle before offering an individualized plan or referral to an appropriate service through *Leeds Let’s Change*[16], a locally provided NHS website that provides information on services that support lifestyle change. The control action is the usual healthy-living assessment that has been implemented in local cardiology services, and which identifies behaviours and offers brief lifestyle advice (including a ‘Leeds Let’s Change’ card).

### Ethical approval

This study was approved by the committee of the National Research Ethics Service for Yorkshire and the Humber (Leeds East) on 12 March 2012. REC Reference number: 12/YH/0086.

### Aims and objectives

The main aim of the study is to assess the feasibility of conducting a definitive trial in terms of recruitment, use and acceptability of the intervention, follow-up at 3 and 6 months, and data collection methods. The following criteria will be used to assess success:

1. Favourable difference shown in referrals and participation.

2. Favourable changes made through self-management.

3. Recruitment target met in time available.

4. Retention rate at 75% or more at 6 months follow-up.

5. Missing data at 10% or less, including 6 months follow-up.

6. Included participants are representative of the target population.

In addition, the study aims to establish suitable procedures for delivering the intervention and conducting assessments and procedures for ensuring recruitment and retention in the study. Finally, the study aims to discover whether using a structured, individualized approach to lifestyle assessment and referral will improve uptake and participation in lifestyle- and behaviour-change interventions;

The study will also examine, qualitatively, the acceptability of the assessment tool to patients in an acute cardiology setting as well as patients’ experiences of making lifestyle changes in order to develop effective recruitment and retention strategies.

The study will have a number of quantitative objectives:

1. To determine how many patients accept referral to a formal lifestyle programme;

2. To determine how many patients participate in a lifestyle-change intervention or initiate self-managed change;

3. To investigate the uptake of lifestyle intervention in relation to subsequent behaviour change and impact on health-related quality of life, mood and social satisfaction;

4. To estimate feasible eligibility, recruitment and refusal rates, and 3- and 6-months follow-up rates;

5. To measure key outcome domains (that is, for completion rates, missing data, estimates, variances and 95% confidence intervals for the difference between the control and intervention groups) for patients including clinical indicators and patient-reported measures of social satisfaction; health-related quality of life; and mood;

6. To synthesize data to inform the sample size of a definitive trial;

7. To determine the acceptability (and factors influencing this) of financial incentives as a method to encourage behaviour change, their pricing and factors influencing this.

## Methods and design

This study is being conducted in cardiology wards at the Leeds Teaching Hospitals Trust with patients admitted to hospital, who are subsequently referred to the cardiac rehabilitation team or discharged with no confirmed diagnosis of cardiac event.

### Trial design

**Figure 1:**
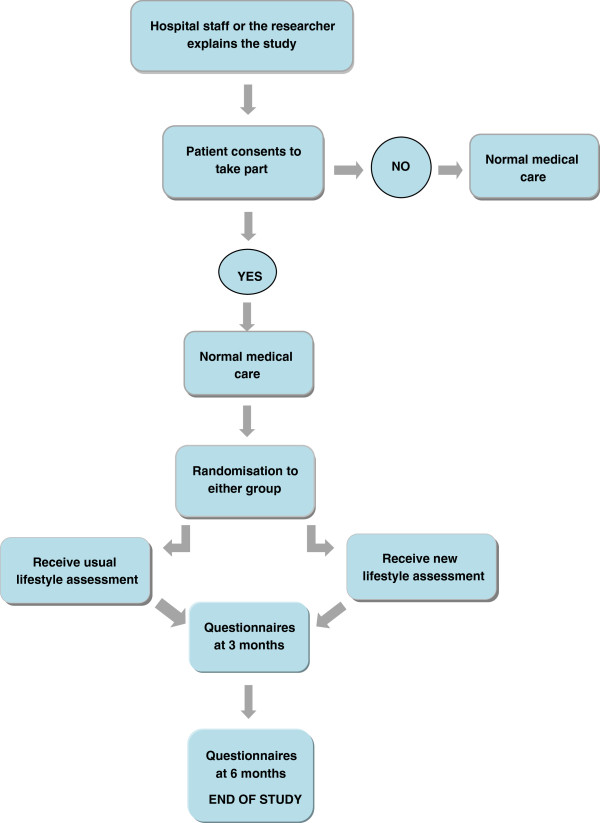
Trial design summary.

The study is designed as a single-centre, randomized, unblinded feasibility trial in an acute hospital setting (Figure 1).

### Incentives sub-study

A sub-study will explore the acceptability and potential usefulness of using financial incentives to help patients overcome motivational and financial barriers to changing their health behaviour. Incentives will not be offered within the trial but we will explore theoretical aspects, such as the pricing and type of incentives offered, and the association of type and magnitude of incentive with perceived difficulty of change. A survey questionnaire specifically designed to explore attitudes to incentives will form part of the study assessments.

### Study conditions

Participants are randomized, using block randomization with randomly varying block sizes, in equal proportions, with no stratification, to receive either usual lifestyle assessment or an enhanced lifestyle assessment using an automated 24-hour telephone randomization service provided by the clinical trials research unit. The usual lifestyle assessment comprises a series of questions that identify lifestyle factors (that is, smoking habits, alcohol consumption, physical activity and weight) that contribute to an increased risk of vascular events. The enhanced lifestyle assessment includes the usual assessment but also contains an evidence-based lifestyle referral assessment based on factors that predict change [2,4]. The aim of this enhanced assessment is to aid healthcare professionals in directing patients toward appropriate lifestyle-change interventions. The assessments are delivered in the hospital ward by research staff before discharge.

### Eligibility criteria

Men or women aged between 40 and 74 years of age at the time of screening, who are willing and able to give written informed consent, and have been admitted to hospital with a diagnosis of acute coronary event, myocardial infarction or symptoms of a cardiac nature will be eligible for inclusion in the study.

Patients who are currently receiving specialist treatment with a primary focus on alcohol, smoking, diet or exercise, or who have no modifiable risk factors for vascular events will be excluded from the study. In addition, patients who will be unavailable for follow-up because they have no fixed abode, are mainly resident abroad, or are currently serving a sentence in prison or have outstanding legal issues likely to lead to imprisonment will be excluded, as will patients who are unable to take part in either intervention using spoken English or are unable to complete the English-language outcome measure tools without assistance.

### Potential sample

Based on figures available at the planning stage, we can estimate that cardiology services in the Leeds Teaching Hospitals Trust receive around 800 admissions per month; on average approximately 300 of these admissions are people who have had an acute myocardial infarction and 150 are people who have experienced an acute cardiac event; the remainder are elective admissions for cardiac-related procedures such as stenting. It is therefore estimated that ten patients per week could take part in the study. This estimate is also based on local knowledge and previous experience of recruiting patients with acute stroke in a similar setting [17].

### Recruitment strategy

All patients admitted to the selected wards are considered potential participants. Potentially eligible patients are identified by research staff from the ward admissions register or by direct referral from ward staff. These patients are approached by the research team or ward staff to determine willingness to participate and an eligibility checklist is completed. Assessment of psychological status and cognitive capacity to participate is made in consultation with ward staff. All patients meeting the eligibility criteria are provided with an explanation of the study and a patient information sheet, and have an opportunity to ask questions. If willing to continue, they are invited to participate in the study and give written consent. Participation in the study does not alter their usual care. Reasons for nonparticipation, date of birth, sex and postcode are recorded whenever possible.

### Recruitment rates, exclusions and generalizability

**Figure 2:**
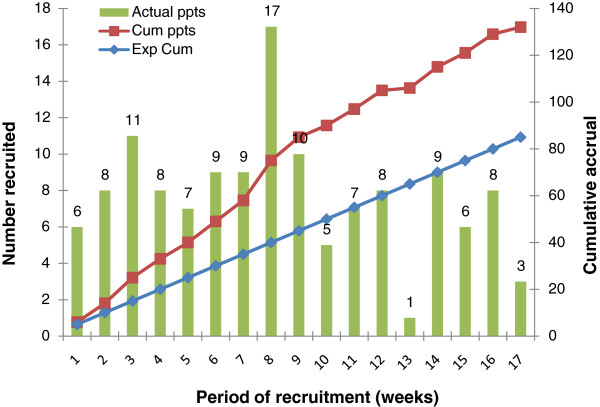
**Recruitment: predicted, weekly and cumulative accrual rates.** Cum, cumulative; Exp, expected.

**Figure 3:**
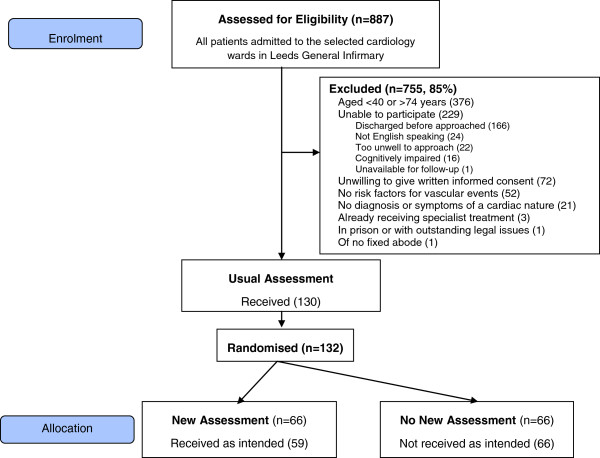
Passage of participants through trial.

The rates of recruitment to the trial are given in Figure 2. Figure 3 provides the numbers of participants at each stage in the trial. Details of the patients are summarized in Table 1.

**Table 1:** Patient characteristics in the screened and randomized samples

	**Screened (*n *= 887)**	**Randomized (*n *= 132)**
Age (years)		
Up to 35	32 (3.6)	0 (0.0)
35 to 40	13 (1.5)	0 (0.0)
40 to 50	82 (9.3)	25 (18.9)
50 to 60	149 (16.9)	44 (33.3)
60 to 70	163 (18.5)	44 (33.3)
70 to 75	107 (12.1)	19 (14.4)
75 to 80	130 (14.7)	0 (0.0)
80 to 85	122 (13.8)	0 (0.0)
85 and over	85 (9.6)	0 (0.0)
Male	467 (52.6)	81 (61.4)
Female	420 (47.4)	51 (38.6)
**Reasons for admission**		
Symptoms	718 (81.0)	116 (87.9)
Acute coronary event	97 (10.9)	15 (11.4)
Elective procedure	26 (2.9)	0 (0.0)
Atrial fibrillation	10 (1.1)	1 (0.8)
Other	6 (0.7)	0 (0.0)
Unknown	30 (3.4)	0 (0.0)

Data are *n* (%). Age is available for 883 screened patients.

#### Study measures

##### Visit schedule

This is a longitudinal study with two follow-up points. Data (Table 2) are collected at baseline (during the hospital admission), then at three and six months following randomization, plus or minus two weeks. Follow-up data are collected by email, post and telephone, or during a home visit if required. Recommended techniques, such as enclosing stamped, addressed envelopes for returning questionnaires and sending reminders, will be used to maximize response rates [18].

**Table 2:** Data collection schedule

**Variable**	**Measurement tool**	**Time-point**				**Type of administration**
		**Screening**	**Baseline**	**3 months**	**6 months**	
Age	Demographics	×				Audit
Sex	Demographics	×				Audit
Postcode	Demographics	×				Audit
Health-related quality of life	EQ-5D		×	×	×	Posted questionnaire
Mood	Clinical Outcomes in Routine Evaluation (10-item version)		×	×	×	Posted questionnaire
Satisfaction	Social Satisfaction Questionnaire		×	×	×	Posted questionnaire
Costs	Valuing Health Change Questionnaire		×	×	×	Posted questionnaire
Uptake	Qualitative			×	×	Interview
Adherence	Qualitative			×	×	Interview
Smoking status	Qualitative		×	×	×	Interview
Alcohol consumption	Qualitative		×	×	×	Interview
Diet	Qualitative		×	×	×	Interview
Physical activity	Qualitative		×	×	×	Interview

*EQ-5D*, European Quality of Life - 5 Dimensions.

##### Assessment tools

Change in lifestyle will be determined by self-reported changes in alcohol consumption [19], smoking, diet and physical activity. Social satisfaction will be measured using the Social Satisfaction Questionnaire, a validated eight-item scale found to be a suitable outcome measure in people with substance use disorders [20]. Subjective wellbeing, psychological problems and functioning will be measured using the Clinical Outcomes in Routine Evaluation (10-item version). [21]. Health-related quality of life will be assessed using the European Quality of Life - 5 Dimensions (EQ-5D) [22]. The EQ-5D is a generic measure of health status, where health is characterized on five dimensions (mobility, self-care, ability to undertake usual activities, pain, anxiety or depression). The EQ-5D visual analogue scale will also be completed. The EQ-5D has been validated in the UK.

#### Health economics

Patients will also complete a questionnaire developed specifically for the trial as part of the incentives sub-study. The Valuing Health Change Questionnaire asks about the perceived difficulty in changing health behaviour, willingness to accept financial (dis)incentives (and minimum acceptable incentive amounts) and some supplementary questions required for the analysis in order to analyze the incentive, including attitudinal and time preference questions.

#### Qualitative data

There are no suitable quantitative measures of uptake, participation and maintenance of behaviour change. Information about participants’ acceptance of lifestyle advice, motivation to change and experience of effecting change will therefore be collected using qualitative methods in a structured interview, which will be conducted at the three and six-month follow-up points. Interviews will be conducted face-to-face or by telephone. The primary endpoints for the trial will be coded from this information and analyzed quantitatively. The interview data will be analyzed using a thematic framework approach and will be used to inform the development of a larger-scale trial.

#### Sample size calculations

We planned to recruit a minimum of 120 patients over four months, randomized equally between intervention and control groups. Assuming that loss to follow-up will be no greater than 25% at 6 months, this will leave 90 patients for our study. This study is designed to assess the feasibility of conducting a definitive trial; because effectiveness is not being evaluated, a formal power calculation would not usually be considered necessary for the primary objectives. However, there is a secondary proof-of-concept element to the study. Based on a two-sample Student’s *t* test with a 2-sided 5% significance level, there would be an 80% power of detecting a standardized effect size of 0.6 in any outcome variable with 90 patients at analysis. As this is larger than the clinically meaningful effect of 0.4, our results will be classed as inconclusive rather than negative if no statistically significant difference is found. We will report 95% confidence intervals and interpret the level of uncertainty based on these.

#### Statistical analysis

The primary analysis will be conducted on the intention-to-treat sample, including all randomized participants in the groups to which they were randomized. Analysis will focus on descriptive statistics and confidence interval estimation rather than formal hypothesis testing. The primary outcome of successful uptake of lifestyle advice is made up of two components: accepting referral or self-referral and participation in lifestyle interventions or self-management. The first is a binary outcome (yes, no) while the second is ordinal (none, initiated, persisted and maintained). Both will be summarized in each group, along with 95% confidence intervals to provide evidence of proof of concept.

A standard CONSORT-approved diagram will depict the flow of patients through the study from screening to analysis (see Figure 3 for enrolment to allocation stages only). This diagram summarizes the feasible eligible, recruitment and refusal rates. It will also summarize the 3- and 6-month follow-up rates. The distributions of the data at baseline, and at 3 and 6 months after randomization will be explored, with unusual values noted and explained. Baseline variables will be summarized as *n* (%), mean (standard deviation) or median (interquartile range), as appropriate, both overall and by randomized group, to characterize the sample and look for any imbalances. Risk factors for vascular events will be summarized, together with the frequency at which patients seek help. Referral choice will then be summarized by randomized group. Responses to the new assessment will be explored, including any changes between first and second assessments. Protocol violations will be reported, including an exploration of the predictors of any missing data. The primary outcomes will be summarized descriptively by behaviour change in relation to risk factors and by health-related quality of life, mood and social satisfaction to assess the relationship between the process and the clinical endpoints. Completion rates and missing item data will be summarized for key secondary outcomes, together with estimates and variances to aid calculation of sample size for a definitive trial.

#### Patient and public involvement (PPI) group

Development of the protocol and patient materials (information sheets and consent forms) benefitted greatly from the involvement of the CLAHRC vascular theme patient and public involvement (PPI) group. The group comprises people with a range of experience of vascular and chronic respiratory conditions, either as patients or as carers. The outcome of the consultation with the PPI group was summarized in an appendix to the full trial protocol (available on request) and submitted to the local research ethics committee. We will undertake further consultation with a wider group of service users during the later phase of the trial, specifically, at the dissemination stage.

## Discussion

Reducing demands on the NHS by giving advice and information to people to enable them to manage and maintain their own health was one of the tenets of the Wanless report [23]. This has been enacted by NHS Trusts across the United Kingdom through the ‘Making Every Contact Count’ initiative by focusing on staff education and training [24]. Less emphasis has been placed on the type of advice, and its outcome in terms of referral to formal interventions, but evidence suggests that lifestyle advice alone has limited success. The assessment of lifestyle is not standardized, although some tools exist and are used in clinical practice. We took evidence from literature reviews and qualitative studies conducted as part of the CLAHRC IMPROVE project and designed a short and simple, systematic assessment that takes into account the barriers and facilitators experienced by patients trying to change their lifestyle [2,4]. We hypothesized that a more individualized approach to assessing lifestyle had the potential to improve uptake and participation in formal lifestyle- or behaviour-change programmes, or improve self-management, to achieve healthy behaviour.

If the results of this preliminary work are positive, and our success criteria are met, we intend to develop a large-scale trial to evaluate the clinical and cost effectiveness of systematized lifestyle assessment on reducing risk factors for cardiovascular disease and improving health status. Other associated effects, such as social functioning and emotional wellbeing, would be considered secondary outcomes. This study is designed to demonstrate that such a trial could be implemented in an acute cardiac setting and to develop an optimized, practicable protocol; secondly, to show that the assessment is viable and can be implemented with patients; and thirdly, to provide an estimate, and a confidence interval, for the efficacy of the assessment, to determine whether a definitive, large-scale trial would be warranted. These aims constitute a balance between, on the one hand, feasibility work and, on the other, proof of concept.

This is the first study of this type of intervention, to our knowledge, in an acute hospital setting, and the first in cardiology. This study paves the way for a larger-scale trial by giving us insight into operational procedures, such as recruitment and follow-up of patients; and the assumptions necessary to calculate the required sample sizes. It also allows us to test and refine methods of data collection and intervention provision. Our larger-scale trial would be multicentre and would require us to consider training hospital staff to deliver the assessment on the ward, as well as introduce different methods of directing patients toward locally available formal programmes. This preliminary work provides a case-study for wider roll-out and generalization and will give a useful indication as to whether there is benefit in approaching different lifestyle behaviours in different ways.

Given current emphasis on lifestyle change as a mechanism for improving health and wellbeing across the population, the significance of the larger-scale trial to which this would lead is important. The difficulties of addressing lifestyle factors and supporting behaviour change are widely acknowledged; therefore, we consider that the results from this study will be of value both to the acute setting and to primary care. A systematic approach to assessment would facilitate audit and provide an indicator of the quality of care. It would also facilitate feedback to clinical commissioners and enable service improvement measures to be implemented where required. The new assessment template has been designed to be quick and easy to use in practice and could, for example, be added to a primary care consultation or form part of a nursing discharge assessment in an acute setting.

## Trial status

The trial is on-going.

## Abbreviations

CLAHRC: Collaboration for applied health research and care; IMPROVE: Improving prevention of vascular events; NICE: National Institute for Health and Clinical Excellence; PPI: Patient and public involvement.

## Competing interests

The authors declared that they have no competing interests.

## Authors’ contributions

KH wrote the study protocol, managed the trial and took the lead in writing the manuscript. REAW designed the statistical analysis and assisted in writing the manuscript. DC implemented the protocol, collected data, refined the study processes and reviewed the manuscript. DM designed the health economics component and contributed to the manuscript. GR provided guidance on clinical aspects of the trial. AJF contributed to writing the protocol, supervised the statistical aspects of the trial and data management and reviewed the manuscript. JYM designed the lifestyle assessment tested in the trial and reviewed the manuscript. AH conceived the trial and contributed to writing the protocol and the manuscript preparation. All authors have read and approved the final manuscript.

## Authors’ information

KMH: BSc, MSc, PhD, Senior Research Fellow.

REAW: BSc, MSc, PhD, Principal Medical Statistician.

DCC: BSc, MSc, Research Officer.

DMM: BA, MSc, Lecturer and Doctoral Student.

JYM: BSc, MSc, PhD, Senior Research Fellow.

GR: MBBS, FRCP, Consultant Cardiologist; Clinical Director, Cardiology and Respiratory Medicine.

AJF: BSc, MSc, Professor of Clinical Trials & Evaluation of Complex Interventions.

AOH: BSc, MBBS, MRCP, MRCPsych, DM, Professor of Liaison Psychiatry.
